# Neurostimulation for Neurogenic Bowel Dysfunction

**DOI:** 10.1155/2013/563294

**Published:** 2013-03-21

**Authors:** J. Worsøe, M. Rasmussen, P. Christensen, K. Krogh

**Affiliations:** ^1^Department of Surgery P, Aarhus University Hospital, Tage-Hansen Gade, 8000 Aarhus, Denmark; ^2^Department of Neurosurgery, Aarhus University Hospital, 8000 Aarhus, Denmark; ^3^Neurogastroenterology Unit, Department of Hepatology and Gastroenterology, Aarhus University Hospital, 8000 Aarhus, Denmark

## Abstract

*Background*. Loss of normal bowel function caused by nerve injury, neurological disease or congenital defects of the nervous system is termed neurogenic bowel dysfunction (NBD). It usually includes combinations of fecal incontinence, constipation, abdominal pain and bloating. When standard treatment of NBD fails surgical procedures are often needed. Neurostimulation has also been investigated, but no consensus exists about efficacy or clinical use. *Methods*. A systematic literature search of NBD treated by sacral anterior root stimulation (SARS), sacral nerve stimulation (SNS), peripheral nerve stimulation, magnetic stimulation, and nerve re-routing was made in Pubmed, Embase, Scopus, and the Cochrane Library. *Results*. SARS improves bowel function in some patients with complete spinal cord injury (SCI). Nerve re-routing is claimed to facilitate defecation through mechanical stimulation of dermatomes in patients with complete or incomplete SCI or myelomeningocele. SNS can reduce NBD in selected patients with a variety of incomplete neurological lesions. Peripheral stimulation using electrical stimulation or magnetic stimulation may represent non-invasive alternatives. *Conclusion*. Numerous methods of neurostimulation to treat NBD have been investigated in pilot studies or retrospective studies. Therefore, larger controlled trials with well-defined inclusion criteria and endpoints are recommended before widespread clinical use of neurostimulation against NBD.

## 1. Introduction

Neurogenic bowel dysfunction (NBD) can be defined as the loss of normal bowel function due to nerve injury, neurological disease, or congenital defects of the nervous system [1]. Symptoms include combinations of fecal incontinence (FI), constipation, abdominal pain, and bloating [2]. NBD is seen in several neurological disorders including spinal cord injury, multiple sclerosis, stroke, Parkinson's disease, and myelomeningocele. It is well documented that NBD has severe impact on quality of life, and by many patients it is considered a greater problem than loss of mobility [2].

Symptoms of NBD vary largely between individuals and do not only depend on the underlying neurological defect but also on other factors like immobility, time since lesion,n and concomitant medication (e.g., spasmolytics, antibiotics, and analgesics). Cerebral insults may impair supraspinal control of defecation resulting in both constipation and FI [3–5]. Spinal cord injury (SCI), multiple sclerosis, and myelomeningocele affect colorectal motility, anorectal sensation and voluntary anal sphincter function [2, 6, 7]. This also results in constipation and fecal incontinence. Parkinson's disease is characterized by striated muscle dystonia also affecting the striated external anal sphincter [8–10]. This results in symptoms of incomplete defecation [11]. Furthermore, depletion of Dopamine producing cells in the colonic wall results in prolonged colonic transit time [12, 13]. Given the very variable pathophysiology of NBD and the common use of medication affecting bowel function, data published on one patient group are not directly applicable to patients with NBD from other diseases and maybe not even to other patients with the same disease. 

Through the last decades a number of treatment modalities have been introduced for the management of NBD. This paper will focus on various methods for nerve stimulation. First the invasive procedures are described chronologically. Second, noninvasive procedures are presented. A systematic literature search was made in Pubmed, Embase, Scopus, and the Cochrane Library using the terms, *neurogenic bowel dysfunction, fecal incontinence, constipation, spinal cord injury, multiple sclerosis, Parkinson's disease, stroke, myelomeningocele, sacral anterior root stimulation, sacral nerve stimulation, posterior tibial nerve stimulation, dorsal genital nerve stimulation, biofeedback, magnetic stimulation, sacral nerve rerouting, Skin-CNS-bladder and artificial somatic-CNS-autonomic reflex pathway. *Only papers in English were included.

## 2. The Sacral Anterior Root Stimulator (SARS)

The sacral anterior root stimulator was developed by Brindley et al. and introduced for the control of neurogenic bladder in patients with SCI [14]. The implant is placed via a laminectomy of L4 to S2. After opening of the dura, the nerve roots from S2 to S4 (or S5) are identified and divided in dorsal and anterior. The dorsal roots are sacrificed, and the anterior roots are placed within the stimulator. Cables are tunneled through to the anterior part of the thorax or abdomen and connected to the receiver block via a separate incision. The receiver is patient controlled through a wireless device [15]. Stimulation of the anterior sacral roots triggers micturition and the sacral deafferentation suppress detrusor overactivity and detrusor sphincter dyssynergia [16]. Because of common innervation, SARS will also stimulate peristalsis in the distal colon and the rectum. Accordingly, SARS can reduce transit time of the rectosigmoid and increase the frequency of defecation [17–19]. However, a disadvantage can be a simultaneous anal sphincter contraction, which will block direct emptying during stimulation. Complications related to the procedure, which can result in explantation, include infection (2%), technical problems with the device (8%), and collection of cerebrospinal fluid around the implant (8%) [20–22].

The clinical indication for SARS is exclusively related to bladder function where the effect is well documented [23–25]. The beneficial effects on defecation and constipation are described less systematically. We identified fourteen papers providing information about bowel function after SARS (Table 1). Many patients use SARS for stimulated defecation either alone or in combination with laxatives. Thus, SARS may alleviate constipation, as most patients treated defecate daily or every other day. Two studies demonstrated significantly reduced time used for defecation after SARS. Furlan et al. compared SARS to Malone antegrade continence enema (MACE) and stoma. The MACE procedure gave the best long-term outcome with respect to bowel function, quality of life, and complication rate [26]. Furthermore, the irreversibility of the sacral deafferentation may limit future treatment options. Selective anterior and posterior root stimulations without the sacral deafferentation have been attempted to avoid this [27]. 

## 3. Sacral Nerve Stimulation

Sacral nerve stimulation (Interstim, Medtronic, MV, USA) was introduced for idiopathic FI in 1995, and subsequently indications have spread to include FI of other etiologies [28]. For SNS, an electrode is placed through a sacral foramen between S2 and S4 (preferably S3). The procedure comprises two stages: a three week percutaneous nerve evaluation test and implantation of the permanent stimulator [29]. Usually, 50% improvement of FI score during the test period is requested before implantation of the permanent stimulator. The permanent pulse generator is placed in a gluteal pocket, where it is accessible for radio programming and replacement. Stimulation parameters are adopted from experience with treatment of urinary symptoms (pulse with of 210 *μ*s, a frequency of 15 Hz, and the amplitude set individually usually in the range between 0.1 V and 10 V).

Schurch et al. measured an early segmental and a late polysegmental reflex mediated by afferent pathways during PNE-test in patients with complete SCI, but none of the patients experienced any effect on symptoms [30]. In contrast, several studies have shown a positive clinical outcome of SNS in patients with incomplete SCI (Table 2). Generally, the number of involuntary bowel movements decreases during stimulation, and the effect remains at medium-term followup [31]. In line with studies of SNS in able-bodied subjects with chronic constipation, SNS may also reduce symptoms of neurogenic constipation. Studies indicate that other patient groups with NBD may benefit from SNS, but evidence is still scarce, and no firm conclusions can be drawn [31–33]. The effects of SNS on anorectal physiology in patients with NBD are conflicting. Some have found that saline retention or anal physiology tests improves, but most find no effect [32–34]. Since SNS has an effect in patients with incomplete but not in those complete SCI, future studies are needed to clarify which spinal pathways are necessary for the clinically important effects of SNS. SNS is a minor invasive procedure which is performed relatively safe. Postoperative complications include infection and lead displacement, and pain at the stimulator site is also reported [34].

## 4. Nerve Rerouting

Nerve rerouting was proposed as early as in 1907 [35]. It was initially developed solely for the treatment of bladder dysfunction, and during the first decades all research was done in animal models [36, 37]. From 1967 results from procedures on patients followed, but all were without certain clinical effect [37–39]. These historical reports were all using nerves rostral to the spinal cord lesion. Since the possible length of a vital anastomosis is very limited, the procedures were restricted to patients with low spinal cord lesions. Furthermore, the use of an anastomosis above the level of lesion carries the risk for further neurological deficits [40].

Therefore, nerve re-routing below the lesion would be attractive, and one such procedure had gained wide spread use among patients with spinal cord injury and myelomeningocele especially in China [41, 42]. This so-called “Xiao procedure” is done by an intradural approach via a L4 to S3 hemilaminectomy. A unilateral nerve anastomosis between anterior (motor) filaments from proximal L5 to the S2 or S3 distal nerve root sheath is established. This allows regrowth of nerve fibers creating a new reflex arch through L5 afferent fibers via the spinal cord to L5 efferent fibers and further via the anastomosis to the S2 or S3 innervation zone [41]. Activation is made by ipsilateral electrical or mechanical stimulation of the L5 dermatome. It is reported that cutaneous stimulation can induce spontaneous bladder emptying. The joint innervation of bladder and the distal colorectum from the sacral segments S2-S4 could form the basis for additional effect on bowel function. The multiple stimuli on a daily basis might facilitate colorectal motility and emptying.

All reports on the procedure have had the main focus on urodynamic parameters [41, 43–45]. No human studies have had objective endpoints for bowel function. One publication in spinal cord injury patients mentions that all patients who regained bladder control also regained bowel control [41]. Another report in two patients indicates improvement in bladder function in both patients and unspecified improvement of bowel function in one patient [43]. Finally a third study describes improved continence for stools and less need for laxatives [45]. The only complications put forward are dorsal flexion paresis in myelomeningocele patients (with preoperative preserved use of L4 and/or L5), intermittent cerebrospinal leakage, and head ache [41, 44, 45].

The documentation for treatment with this method is limited with respect to bladder function and very sparse for bowel function. This has led to the general recommendation to keep this procedure in research protocols before wide spread clinical use [45, 46].

## 5. Noninvasive Modalities

These include peripheral electrical stimulation and magnetic stimulation. The advantages of the methods using external stimulation are that no surgery is required and stimulation can be applied as an outpatient procedure or as home stimulation. The methods are safe with no known complications or side effects apart from possible allergy to plaster electrodes. Furthermore, these are all reversible techniques, which patients may find appealing in light of potential future treatment with neuroregeneration or nerve transplantation.

## 6. Posterior Tibial Nerve Stimulation

The posterior tibial nerve is a mixed sensory-motor nerve with afferent pathways going to the lumbosacral dorsal roots (L4-S3) [47]. Posterior tibial nerve stimulation (PTNS) was first introduced for bladder dysfunction. However, many patients suffer from both urinary incontinence and FI (double incontinence), and some also experienced improvement of FI. Stimulation has been done, either using self-adhesive surface electrodes or by needle electrodes placed distal on the leg near the medial malleolus and a ground surface electrode placed on the ipsilateral leg. In studies with PTNS, pulse width is 0.2 ms, and the frequency is 10 or 20 Hz. The amplitude setting differs from below motor threshold to maximal tolerable current, but generally stimulation amplitude is below 10 mA. Various treatment protocols have been applied ranging from 4 weeks to 12 weeks treatment with scheduled stimulation sessions from daily to every third to fourth day. Several studies investigating the effect of PTNS in various nonneurogenic patients have indicated that FI is reduced [48–52]. Mentes et al. examined two SCI patients with an incomplete lesion and reported improvement of the Wexner incontinence score [53]. A large randomized multicenter trial compared PTNS using self-adhesive surface electrodes with sham stimulation in neurogenic patients with incomplete lesions. The specific data for the neurogenic patients were not presented, but overall PTNS had no significant effect on neither the number of FI episodes nor anorectal physiology [54]. 

## 7. Dorsal Genital Nerve Stimulation

Dorsal genital nerve (DGN) stimulation has also been investigated as a method against NBD based on experiences from urology. The pudendal nerve is a mixed sensory-motor nerve originating from S2 to S4. The dorsal genital branch carries afferent fibers, and it is easily accessible peripherally. The effect on anorectal motility has been investigated in pilot studies among patients with complete supraconal SCI, but results have been conflicting. In one study rectal compliance increased, but in the other it decreased during acute stimulation [55, 56]. There are no published studies on the clinical effects of DGN in patients with NBD. Three studies have, however, demonstrated that DGN stimulation can reduce fecal incontinence in patients with pudendal neuropathy and idiopathic FI [57–59]. Stimulation is performed using a battery powered handheld stimulator (Itouch Plus, TensCare, Epsom, UK). Monophasic square constant current pulses with a pulse duration of 200 *μ*s at a pulse rate of 20 Hz are used. One electrode (dimensions: 20 × 10 mm, Neuroline 700, Ambu, Ballerup, Denmark) is placed on the clitoris as a cathode, and a second electrode (diameter: 32 mm, PALS Platinium, Axelgaard, Lystrup, Denmark) is placed 2-3 cm lateral to the right labia major.

## 8. Magnetic Stimulation

Magnetic stimulation is based on Faradays law (induction of a current by a magnetic field and induction of a current in a secondary circuit close to a primary current-carrying circuit). In 1981 the first magnetic stimulation of a peripheral nerve in a human was undertaken and a few years later magnetic stimulation of the brain followed [60, 61]. Magnetic stimulation has also been applied to alter colonic motility in patients with neurological disorders. In most cases stimulation was applied on the back above the lumbosacral region. Morren et al. and Shafik demonstrated increased rectal pressure during stimulation in subjects with SCI [62, 63]. Using both transabdominal and lumbosacral stimulations Lin et al. demonstrated a significant increase in rectal pressure during acute stimulation. This was followed by reduced colonic transit time with five weeks stimulation [64]. Tsai et al. applied stimulation during three weeks for 20 minutes twice daily in 22 SCI patients including both subjects with supraconal and conal/cauda lesions, and found a significant decrease in colonic transit time and improved bowel dysfunction score [65]. The use of laxatives, the number of unsuccessful attempts at evacuation, and the feeling of incomplete evacuation all decreased. Chiu et al. investigated sixteen patients with Parkinson's disease using a similar protocol [66]. The colonic transit time significantly decreased and rectal emptying, assessed with defaecography, improved significantly. The neurogenic bowel dysfunction score also improved after three weeks stimulation and this was sustained at three months follow-up.

Whether magnetic stimulation could also treat fecal incontinence in patients with neurological disorders it remains to be explored. A randomized study compared magnetic stimulation and sham stimulation in healthy able subjects [67]. The appearance of high-pressure contractions and high pressure propagated contractions provoked by Bisacodyl instillation was significantly delayed during stimulation. Accordingly, the perception of urgency tended to be lower with stimulation following Bisacodyl instillation. After Bisacodyl administration, a catheter was expulsed significantly slower during stimulation than with sham stimulation.

## 9. Discussion

Neurogenic bowel dysfunction is a major problem to a large number of patients worldwide. It restricts participation in social activities and has significant negative effect on quality of life. Most patients respond to standard treatment with dietary advice, oral laxatives, digital rectal stimulation, suppositories, or mini enema. Others benefit from the use of transanal colonic irrigation [68]. For some of those not responding to conservative treatment the Malone antegrade colonic enema through an appendicostomy is an option while others are better treated with a colostomy [69, 70].

Even though several treatment modalities are available, many patients with neurological disorders continue to have severe symptoms. Therefore, new treatments should be explored, developed, and evaluated. As shown in the present paper, stimulation of nervous pathways affecting bowel function has been the subject of a large number of studies. Unfortunately, the majority of publications have been pilot studies with few patients. Others were retrospective, and followup was often very short. In most studies no sham stimulation was performed, and the placebo effects may have been important. The bladder and the distal bowel have common innervation from the sacral spinal cord. Therefore, methods applied for neurogenic bladder dysfunction affect colorectal function as well. Several publications have had the main focus on bladder function, wherefore bowel function is less systematically described, and endpoints for assessment of bowel function poorly defined. 

In spite of the previous reservations, neurostimulation holds promise for future treatment of NBD. Patients with bowel problems secondary to neurological lesions or disease are an extremely heterogeneous group, and it is likely that future treatment with nerve stimulation should reflect the underlying pathology. This calls for better understanding of the mechanisms of action for various types of neurostimulation. Sacral anterior root stimulation works by acute stimulation of the efferent nerve root controlling anorecal motility. When the root stimulation is turned off, the effect vanishes. This allows individual timing of stimulation and makes defecation predictable to some patients. Since many patients with NBD have the dual problem of constipation and fecal incontinence, reliable and predictable defecation is of paramount importance. As opposed to SARS, SNS is thought to work by chronic efferent and afferent stimulation. Neuromodulation of afferent input could explain why SNS reduces symptoms of both incontinence and constipation [71, 72]. Specific alteration of transit in the right colon and activity in vagal projections demonstrated using PET scan indicate that SNS modulate supra sacral and cerebral neural pathways [73, 74]. The effect of SNS in complete SCI patients is generally unexplored. If SNS is effective in complete SCI the mechanism of action would be primarily by efferent stimulation. Modulation of supraconal or even supraspinal pathways may explain why SNS may not be effective in patients with a complete SCI. 

With peripheral nerve stimulation, an electrical pulse or a magnetic field is applied to an afferent neural pathway and through synaptic transmission modulates other neural pathways innervating the bowel. Stimulation protocols exert a scheduled number of sessions, and as patients usually do not defecate during the short stimulation periods, treatment is based on the assumption of an effect lasting beyond stimulation. Whether such long-lasting modulation of neural pathways and bowel function is possible it is not fully established. A comparison of transcutaneous stimulation with surface electrodes and percutaneous stimulation with needle electrodes also remains to be studied in neurogenic patients.

If nerve rerouting is feasible for the treatment of NBD symptoms, it awaits clarification. The current lack of scientific evidence of nerve rerouting regarding bowel function restricts the use of this modality to research protocols.

## 10. Conclusion

Neurostimulation represents ways to reestablish neurogenic control and thereby alleviate NBD symptoms. While several studies have demonstrated proof-of-concept of these treatments, larger randomized studies are lacking, and long-term effects should be evaluated. Such studies are mandatory to define the indications for each of the techniques and to clarify the right place for each modality in a treatment algorithm. To optimize the procedures various stimulation parameters should be compared, and for methods based on intermittent stimulation the frequency and duration of treatment sessions should be defined. At present, patients and clinicians do not have the necessary information to choose nerve stimulation against NBD and certainly not to select between various methods. In the opinion of the authors, neurostimulation for NBD should be restricted to scientific protocols until further evidence of effects is provided by randomized trials. Such trials should probably be performed in international multicentre studies.

## Figures and Tables

**Table 1:** Results of sacral anterior root stimulation in patients with spinal cord injury and bowel dysfunction. As illustrated endpoints vary between studies with some lacking well-defined endpoint for bowel function.

	Patients (*n*)	Median followup (years)	Patients using SARS for defecation (*n*)	Frequency of defecation with use of SARS (median or compared to preoperative)	Time used for defecation with use of SARS (median or compared to preoperative)	Patients with complete evacuation using SARS (*n*)
Binnie et al. [18]	10	2.6	10	5.5/week		—
Brindley [75]	50	3.8	—	Increased		Improved
Brindley and Rushton [76]	50	6.8	27	—	Decreased	—
Creasey et al. [77]	17	1	17	4/week	12 min*	
Egon et al. [78]	68	5.4	51	—	Reduced in all	21
Kerrebroeck et al. [24]	184	—	110	—	—	36
MacDonagh et al. [19]	12	2.2	12	8.3	28.9 min*	6
Madersbacher and Fischer [79]	7	0.5–2	2	—	—	1
Kutzenberger et al. [80]	440	6.6	401	4.9/week		
Sarrias et al. [81]	7	—	7	—	—	4
Vall$\overset{`}{\text{e}}$s et al. [82]	18	12	18	8.2/week	Decreased	8
Varma et al. [17]	5	1.2	—	—	—	Not improved
Vastenholt et al. [83]	37	7	22	—	Decreased	
van der Aa et al. [84]	37	0.25–13	27	Increased	Decreased	

**Table 2:** Results from sacral nerve stimulation in patients with neurogenic bowel dysfunction.

	Etiology (*N*)	Successful PNE-test (%)	Symptoms baseline	Symptoms followup (months)
Ganio et al. [31]	SCI trauma (2) SCI surgery (4) Spastic paresis (1) Tethered cord (1) Pelvic nerve lesion (1) Poliomyelitis (1)	6 (60)	Median 2 incontinence episodes per week	

Gstaltner et al. [85]	Cauda equina (11)	8 (73)	Median WexInc. 15	Median WexInc. 5

Holzer et al. [33]	SCI surgery (17) Myelomeningocele (4) Friedrich's ataxia (1) MS (1) Diabetic neuropathy (1) Spinal insult (1)	18 (72)	Median 7 incontinence episode/3 weeks	Median 2 incontinence episode/3 weeks (35)

Jarrett et al. [34]	Disc prolapse (6) Trauma (4) Spinal stenosis (1) Neurosurgery (2)	12 (92)	Mean 9.33 incontinence episodes per week	Mean 2.39 incontinence episodes per week (12)

Lombardi et al. [86]	Spinal cord injury (39)	23 (59) 12 constipation 11 incontinence	Mean WexCon. 19.91 (12) Mean WexInc. 13.09 (11)	Mean WexCon 6.82 (44.3) Mean WexInc. 4.91 (46)

Rosen et al. [32]	Spinal cord injury (6) Spinal cord surgery (4) Meningomyelocele (2) Multiple sclerosis (1) Friedreich's ataxia (1) Spinal stroke (1)	11 (73)	Median 7 incontinence episode per 3 weeks	Median 2 incontinence episode per 3 weeks (15)

WexInc: Wexner fecal incontinence score, WexCon: Wexner constipation score.
